# Development of a Novel ssDNA Sequence for a Glycated Human Serum Albumin and Construction of a Simple Aptasensor System Based on Reduced Graphene Oxide (rGO)

**DOI:** 10.3390/bios10100141

**Published:** 2020-10-14

**Authors:** A-Ru Kim, Yeongmi Choi, Sang-Heon Kim, Hyun-Seok Moon, Jae-Ho Ko, Moon-Young Yoon

**Affiliations:** 1 Department of Chemistry and Research Institute of Natural Sciences, Hanyang University, Seoul 04763, Korea; kimr2122@hanyang.ac.kr (A.-R.K.); onomu@hanyang.ac.kr (Y.C.); konasi2@naver.com (S.-H.K.); 2 Sungsan Eng Co.ltd., Gumi 39377, Korea; anoldmoon1@nate.com (H.-S.M.); jhko001@daum.net (J.-H.K.)

**Keywords:** diabetes, glycated human serum albumin, SELEX, aptamer, reduced graphene oxide

## Abstract

Diabetes is one of the top 10 global causes of death. About one in 11 global adults have diabetes. As the disease progresses, the mortality rate increases, and complications can develop. Thus, early detection and effective management of diabetes are especially important. Herein, we present a novel glycated human serum albumin (GHSA) aptamer, i.e., GABAS-01, which has high affinity and specificity. The aptamer was selected by reduced graphene oxide-based systematic evolution of ligands by exponential enrichement (rGO-based SELEX) against GHSA. After five rounds of selection through gradually harsher conditions, GABAS-01 with high affinity and specificity for the target was obtained. GABAS-01 was labeled by FAM at the 5′-end and characterized by measuring the recovery of a fluorescence signal that is the result of fluorescence quenching effect of rGO. As a result, GABAS-01 had low-nanomolar Kd values of 1.748 ± 0.227 nM and showed a low limit of detection of 16.40 μg/mL against GHSA. This result shows the potential application of GABAS-01 as an effective on-site detection probe of GHSA. In addition, these properties of GABAS-01 are expected to contribute to detection of GHSA in diagnostic fields.

## 1. Introduction

Diabetes is a group of metabolic diseases in which high blood sugar level persists for a long time. Diabetes is caused by dysfunction of insulin production, leading to hyperglycemia, and typically includes type 1 diabetes and type 2 diabetes [1]. Type 1 diabetes occurs when the immune system in the body attacks and destroys the pancreatic cells that produce insulin [2]. Thus, insulin secretion is blocked, and insulin action is hindered when sugar builds up in the bloodstream. Type 1 diabetes is called ‘insulin-dependent diabetes mellitus’, and type 2 diabetes is called ‘non-insulin-dependent diabetes mellitus’. Type 2 diabetes is the more common type of diabetes and is caused by lifestyle and genetics [3,4]. In this type, cells develop a resistance to the action of insulin to result in impaired insulin secretion. As the pancreas does make not enough insulin to overcome this resistance, insulin actions are relatively reduced, and the blood sugar level is increased [4,5,6].

Diabetes mellitus was one of the top 10 global causes of death in 2016 [7]. According to the International Diabetes Federation’s diabetes atlas, 285 million people (between the ages of 20 and 79 years) suffered from diabetes in 2010. In 2019, the number of patients increased to 463 million with a 4.2% mortality rate. This trend is increasing continuously, and it is expected that 578 million people will have diabetes in 2030 [8]. Diabetes can lead to severe long-term complications. The major complications are related to nerves (neuropathy), kidneys (nephropathy), eyes (retinopathy), and blood vessels in the heart [9,10]. These complications lead to increased mortality risk, which is especially high in young-onset type 2 diabetes [10,11]. Hence, early detection and effective management of diabetes are crucial.

The American Diabetes Association (ADA) has recommended screening guidelines for diabetes since 2009, and the World Health Organization (WHO) supports these guidelines [12,13]. According to their reports, a fasting plasma glucose (FPG) test and an oral glucose tolerance test (OGTT) can be used to measure the blood sugar level. The FPG test is faster and easier than other tests. However, it requires overnight fasting and measures the blood sugar level at the time of blood sampling. The OGTT is more sensitive than the FPG test, but it is less convenient to manage. It requires fasting for more than eight hours and measures blood glucose immediately before and two hours after a person drinks a liquid containing 75 g of glucose dissolved in water [12]. The criterion for diabetes diagnosis is an FPG level ≥ 126 mg/dL (7.0 mmol/L) or a 2 h plasma glucose level ≥ 200 mg/dL (11.1 mmol/L) during an OGTT. The other test that the ADA recommended is the glycated hemoglobin (HbA1c) test. This test does not require fasting and provides a reliable measure to monitor and manage chronic glycemia because it detects the average blood sugar level for the past 2–3 months [14]. However, it has several limitations. First, it is not suitable for detecting short-term changes in blood sugar. Additionally, for pregnant women and patients with disorders like hemolytic anemia or thalassemia, it is unreliable. Additionally, because the HbA1c test cannot distinguish between fasting blood glucose and postprandial blood sugar levels, it is difficult to determine if additional adjustment is required [15]. However, these drawbacks can be overcome by using glycated human serum albumin (GHSA) [16,17].

Albumin is the most abundant protein in both human blood and major organs. Glycated albumin refers to albumin that is bonded by non-enzymatic glycation. It measures the average blood sugar level over the past 2–3 weeks, which is a shorter period of time than the HbA1c test [18]. GHSA allows for a more accurate diagnosis and treatment; therefore, it can be used as a more accurate marker for diabetes mellitus.

Aptamers are short single-stranded oligonucleotides (ssDNA or RNA). As the aptamers are folded into a specific three-dimensional structure, they have high affinity and selectivity against the target molecules. They are developed via systematic evolution of ligands by exponential enrichment (SELEX), which was first reported in 1990 [19,20]. The target range of SELEX is wide, including small molecules, proteins, immunogens, organisms, and even whole cells [21,22,23,24,25,26,27,28]. Compared with antibodies, aptamers have several benefits, such as lower cost, smaller size, lower immunogenicity, and easier synthesis and chemical modifications [29,30]. Additionally, aptamers are stable in harsh conditions. Hence, aptamers have been used in biotechnology, biosensing, and biomedicine [31,32,33].

To obtain specific aptamers, SELEX technology has been developed. The traditional SELEX methods need a target immobilizing step [34,35]. However, some cases of immobilizing methods are very complex and time-consuming processes. Moreover, these methods may change the conformations of the targets and occupy a binding site of the targets. To solve this problem, we performed SELEX using reduced graphene oxide (rGO) system. Generally, graphene oxide (GO) can adsorb single-stranded oligonucleotides through π-π stacking interactions and it requires simple detachment methods to separate the adsorbed single-stranded oligonucleotides from graphene oxide [36,37]. The detachment methods need addition of complementary sequences or other molecules such as proteins that lead to conformational changes, because the interactions between the single-stranded oligonucleotides and GO are the result of the exposed phosphate backbone [36,37,38,39].

In this work, graphene was also used as a substrate for nonspecific adsorption of fluorescent dye-labeled aptamers in SELEX process. Graphene has a linear band dispersion around the corners of its Brillouin zone (BZ) and a nearly constant optical absorption; thus, optically excited species can be quenched by resonance energy transfer via excitation of electron-hole pairs in the graphene [40,41,42,43,44]. Besides, using this principle, rGO system was used to characterize binding affinity and specificity of aptamers against the target as well.

In this research, we propose a novel sequence of DNA aptamers with high binding affinity for GHSA and suggest that the screening method is important for constructing the sensor system.

## 2. Materials and Methods

### 2.1. Materials

The single-stranded DNA library and primers were synthesized by Bioneer (Daejeon, Korea). pfu DNA polymerase was purchased from Biofact (Daejeon, Korea). Restriction enzymes and DNA ligases were purchased from Takara Bio (Shiga, Japan). Reduced graphene oxide was purchased from Graphene Supermarket (Calverton, NY, USA). Other chemicals were obtained from commercial sources with high quality.

### 2.2. Construction of the ssDNA Library and Primers

The methodology of constructing ssDNA library and primers is similar to previous research [45]. To develop a specific aptamer that binds to GHSA, the ssDNA library was synthesized with 30 central random bases flanked by two sets of 15 conserved bases. The library, which consisted of 60 bases (5′-ATGCGGATCCCGCGC-(N)30-GCGCGAAGCTTGCGC-3′), was used for the template in the first round of SELEX. Primer sites were contained at the BamH I site for the forward primer (5′-ATGCGGATCCCGCGC-3′) and at the Hind III site for the reverse primer. To amplify the template DNA library, 100 μM of forward primer and 10 μM of reverse primer were used for the asymmetric polymerase chain reaction (PCR). The PCR product was electrophoresed on 2.5% agarose gel for confirmation. Next, the crush and soak method was performed to extract the ssDNA library. Additionally, the PCR product was electrophoresed on 12% native gel and stained by ethidium bromide (EtBr) to isolate ssDNA from double-stranded DNA (dsDNA). The ssDNA band was cut out, pulverized, and extracted with crush and soak buffer (500 mM NH_4_OAc, 0.1% SDS, 0.1 mM ethylenediaminetetraacetic acid (EDTA)).

### 2.3. Aptamer Screening with Reduced Graphene Oxide

The methodology of screening used here is similar to one described previously [45]. In vitro SELEX against GHSA was performed with a reduced graphene oxide (2 mg/mL in dH_2_O) system. We performed five rounds of screening, and human serum albumin (HSA) was used as a counter target in each round to obtain aptamer sequences that bind to GHSA specifically.

First, 200 pmol of the ssDNA library pool, 50 μL of the rGO solution (2 mg/mL), 500 μg of the counter target diluted in distilled water, and 40 μL of 5X PBS were mixed and incubated at room temperature for 60 min to immobilize the ssDNA library onto the rGO and eliminate candidates that can bind to HSA. The total reaction volume was 200 μL and pH of mixture is 7.4. The mixture was centrifuged at 14,000 rpm for 20 min, and the supernatant was removed. Next, the rGO-bound ssDNA library was washed three times with 200 μL of 1X PBS in pH 7.4 to remove unbound and weakly bound sequences, followed by another round of centrifugation. After that, for positive selection, 500 μg of GHSA diluted in 200 μL of 1X PBS in pH 7.4 was added and incubated at R.T. for 60 min to elute the target binding aptamers from rGO. When the aptamer was eluted by the target, the binding time and buffer condition for reaction were regulated to select more specific aptamers.

### 2.4. Sequence Analysis

After five rounds of selection, cloning was performed to analyze the selected aptamers. The aptamers were amplified to dsDNA by PCR. The amplified dsDNA product was cut using restriction enzymes (Hind III and Bam HI) and ligated into the pET28a (+) expression vector with T4 DNA ligase. Ligation was carried out at 16 °C overnight and transformed into *Escherichia coli*. The recombinant *E. coli* was cultured overnight at 37 °C in LB medium. Then, the DNA was extracted using a plasmid mini extraction kit (Bioneer, Daejeon, Korea) and sequenced by Macrogen Inc. (Seoul, Korea).

### 2.5. Characterization of the Aptamer with rGO

The obtained aptamer was labeled by FAM (6-carboxyfluorescein) at the 5′-end and characterized using the fluorescence recovery signal. Each experiment was repeated three times.

In the binding affinity test, each concentration of aptamer was incubated with 12.5 μg of GHSA diluted in 100 μL of 1× PBS for 30 min to induce aptamer-protein binding. We added 100 μL of rGO diluted in 1× PBS in pH 7.4 (0.5 mg/mL) to eliminate unbound aptamer residues. We performed centrifugation at 14,000 rpm for 20 min to separate supernatants. Each of the reaction samples was transferred to a 96-well opaque plate, and the fluorescence signal was measured.

The specificity test for other serum albumins was performed using 125 nM of aptamer. The processes and the conditions of reactions were the same as those used for the binding affinity test. Human serum albumin (HSA) and bovine serum albumin (BSA) were used for the specificity test. The experiments of these two controls were undertaken at the same molarity as the GHSA.

The detection limit of the aptamer was measured using various amounts of the target. The concentration of aptamer was 125 nM, and the concentrations of target were 0, 1.95, 3.91, 7.81, 15.625, 31.25, 62.5 and 125 μg/mL. The reaction volume was 100 μL. The processes and reaction conditions were the same as those stated above. The limit of detection (LOD) value was calculated by the official formula established by the International Union of Pure and Applied Chemistry (IUPAC):

LOD = 3 × SD/slope

Here, SD represents the standard deviation at a minimum point, and the slope is obtained from the fitted graph.

### 2.6. Target Detection in Human Serum Solution

Detection of GHSA in human serum solution which is diluted 1/1000 in PBS was achieved through the fluorescence aptasensor system with rGO. The concentrations of GHSA for the tests were 15.625, 31.25, 62.5 and 125 μg/mL.

The analysis process was performed in the same way as the LOD determination.

## 3. Results

### 3.1. Development of Aptamers against GHSA via rGO-SELEX

To isolate the specific aptamers against GHSA, we performed rGO-based SELEX. The ssDNA library was incubated with rGO. The DNA bases were bound to the polycyclic aromatic rings of the graphene surface via π-π stacking interactions [36,37]. The non-bound sequences on rGO and the bound sequences with the counter target were separated by centrifugation and removed. Next, the sequences adsorbed on the rGO surface were eluted by introducing the target compound, amplified by PCR, and used for the next round of selection. Each round of selection proceeded in gradually harsher buffer conditions and shorter elution times to obtain more specific aptamers for the target compound. In Table 1, binding means aptamer-graphene interaction and elution means aptamer–target interaction. The binding buffer is PBS.

**Figure 1:**
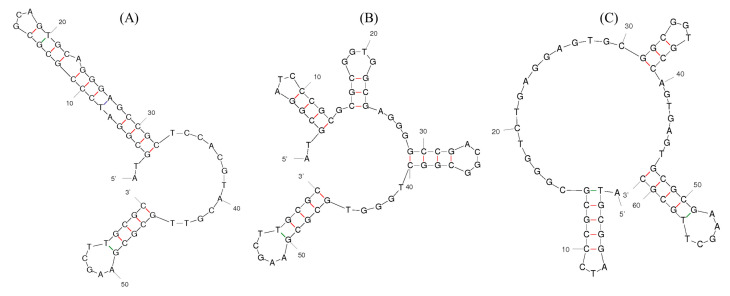
The secondary structures of candidate aptamers: (**A**) GABAS-01, (**B**) GABAS-02, and (**C**) GABAS-03.

The amplified aptamer sequences were inserted into the pET-28a vector and inoculated. After *E. coli* was cultured overnight at 37 °C Development of Aptamers against GHSA via rGO-Selex in the LB medium, three sequences were selected based on frequency and labeled (Table 2). The secondary structures of all selected aptamers were predicted by the M-fold program and exhibited different structure patterns (Figure 1).

### 3.2. Binding Affinity of the Selected Aptamer

**Figure 2:**
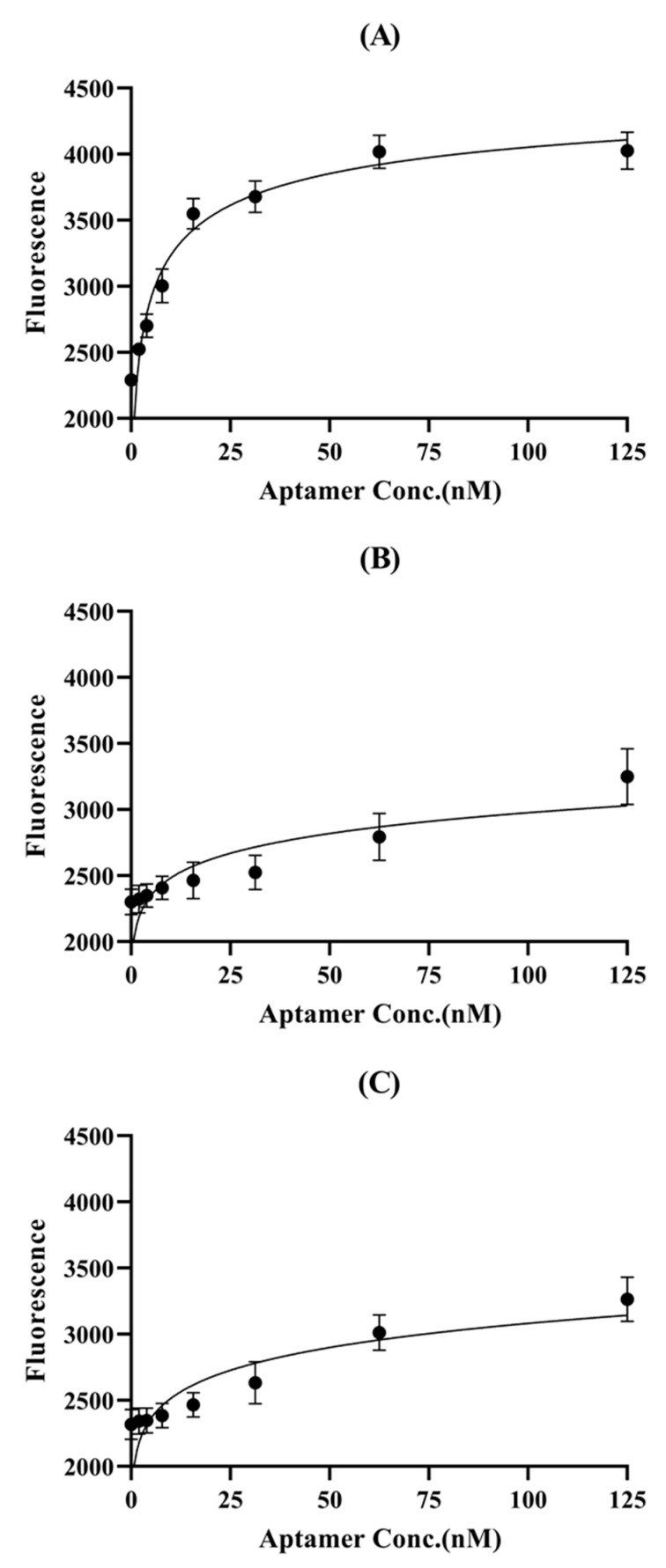
Binding affinity test of aptamer candidates with fluorescence signals. The K_d_ value of (**A**) GABAS-01 is 1.748 ± 0.227 nM, (**B**) GABAS-02 and (**C**) GABAS-03 were not determined.

The fluorescein amidite (FAM)-labeled glycated human serum albumin binding aptamers (GABASs) were characterized based on the fluorescence recovery signal. First, the aptamer was reacted with the target protein, and rGO was added to eliminate aptamer residue. When the aptamer was bound to the surface of rGO, the fluorescence signal was decreased. As shown in Figure 2, the fluorescence signal of GABAS-01 was saturated at near 31.25 nM, while the signals of GABAS-02 and GABAS-03 were not saturated in the same concentration. This experimental process was repeated three times, and the K_d_ (dissociation constant) value of GABAS-01 was determined to be 1.748 ± 0.227 nM and the K_d_ values for the other two aptamers, GABAS-02 and GABAS-03, were not determined exactly in this experiment.

### 3.3. Specificity Test

**Figure 3:**
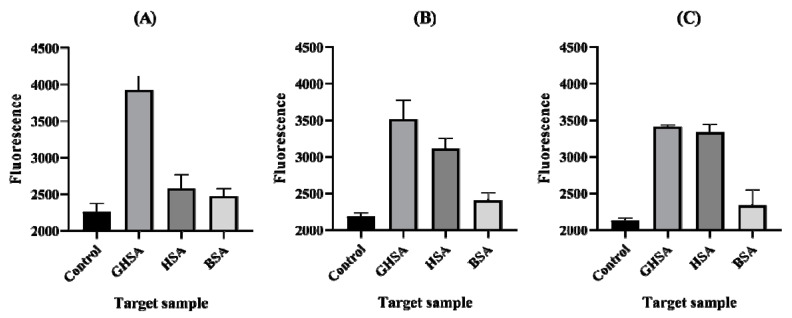
Specificity test of aptamer candidates with fluorescence signal. Albumins GHSA, HSA, and BSA were used to compare the signals: (**A**) GABAS-01, (**B**) GABAS-02, and (**C**) GABAS-03.

Specificity test was performed using human serum albumin (HSA) and bovine serum albumin (BSA) to confirm the specificity of the aptamer in the rGO-based aptasensor. HSA and BSA were treated with the same molarity of GHSA as used in the fluorescence sensing step. The results showed lower fluorescence intensities than GHSA (Figure 3).

### 3.4. LOD Determination of GABAS-01

**Figure 4:**
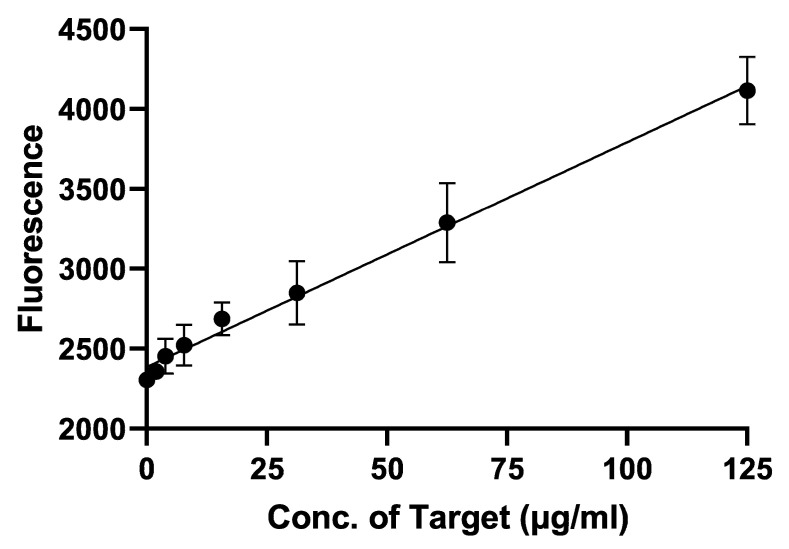
The detection limit of the rGO-GABAS-01 aptanano sensor system. A linear fluorescence response was observed in the range of 0–125 μg/mL GHSA. The limit of detection (LOD) was determined to be 16.40 μg/mL using the official formula from the IUPAC (LOD = 3 × SD/slope).

From the result of binding affinity and specificity test, we selected GABAS-01 for the aptasensor based on graphene-fluorescence system. The LOD value was determined using nearly the same process as described above. The fluorescence signal was measured to calculate the LOD; this depended on the concentration of GABAS-01. As the concentration of GABAS-01 increased, the fluorescence signal increased (Figure 4). A linear graph was obtained, and the LOD was determined to be 16.40 μg/mL, according to the recovery signal in the range of 0–125 μg/mL GHSA. The R-squared value was calculated to be 0.9901, indicating a quite accurate result.

### 3.5. GHSA Detection from Human Serum Solution

We confirmed from Figure 3 that GABAS-01 can bind with specificity. However, this result just shows when only one protein is in buffer but does not indicate when GHSA is mixed with other proteins. Therefore, we did a spike test in human serum solution diluted in 1x PBS in pH 7.4 (Table 3). The concentrations of the spiked solution were 15.625, 31.25, 62.5 and 125 μg/mL. The recovery rate was almost the same with binding buffer, and the maximum value of the relative standard deviation was approximately 2.7%, although the sample was a detergent solution (Table 3). Thus, detection of GHSA was achievable in samples using GABAS-01. This approach could provide the basis of a fluorescence based detection system.

## 4. Discussion

According to previous articles, after binding to the target, aptamer conformation is changed, which weakens the π-π stacking interactions between aptamers and rGO [45,46,47,48,49]. Our group confirmed this by analyzing the high fluorescence signal intensity of the aptamer (Figure 2, Figure 3 and Figure 4).

The K_d_ value of GABAS-01 indicates that GABAS-01 has a high binding affinity for GHSA. This value is much lower than those of the other aptamers for glycated human serum albumin, which displayed affinities in the micromolar range [38].

According to the results from the specificity test, we confirmed that GABAS-01 specifically bound to GHSA, but the others did not demonstrate specificity to the GHSA. The result of specificity test for GABAS-01 also showed the higher signal of GHSA than HSA, though the difference of each protein structure was very slight. Albumin converts into glycated albumin by glycation, which is a non-enzymatic spontaneous reaction where a reducing sugar is added to a free amino group (typically lysine or arginine) present within the proteins. This is also called the Maillard reaction [16,50,51,52]. The possible sites for the reaction are Lys-199, Lys-276, Lys-281, Lys-378, Lys-439, Lys-525, and Lys-545 in human serum albumin, and Lys-525 has higher participation than other lysine residues in the overall glycation processes [53,54,55]. BSA, with 583 amino acids, has high sequence homology to human serum albumin, with a 76% amino acid similarity [55,56]. However, the 24% difference creates a subtle structural difference. Based on previous research and our results, we estimate that GABAS-01 can distinguish the structural difference, and the main binding site of this aptamer would be near the site of the lysine residue.

GHSA is a diagnostic marker for diabetes mellitus. It is used to assess the changes of blood glucose levels over the last 1–2 weeks. It is able to confirm blood glucose changes over a more recent period than the glycated hemoglobin test. The ratio of GHSA is currently confirmed by an enzymatic method based on the colorimetric system. This system can be analyzed with the naked eye and shows better efficiency with the help of a photometer. However, this method usually has relatively low sensitivities (Table 4). Aptasensor systems based on fluorescence have also been developed [38]; this graphene methodology is almost the same as our system and the LOD of this aptamer system is 50 μg/mL. Our system’s LOD is 16.40 μg/mL and it is more sensitive. This discrepancy in LOD value is likely caused by differences in screening strategy. As mentioned above, target immobilizing methods may change the conformations of the targets and occupy a binding site of the targets. The platform to immobilize the target which like beads, plates, or etc., shield immobilized the site of the target from aptamer. This makes the blind site of target able to interact stronger with aptamer candidates or aptamer candidates which can only recognize the immobilized target. Therefore, sometimes, we can find aptamer candidates that can bind to target with high affinity and specificity in immobilized condition but cannot bind in other conditions. Therefore, the screening method of the aptamers are important. Our results show that GABAS-01 may be used as a novel, integral part of an aptasensor with a signal transduction system; this could lead to a simple, cost-effective, and time-saving system.

## 5. Conclusions

This article identifies a novel GHSA binding aptamer through library screening that can be used for detection of GHSA among similar proteins. In this paper, we show that differences in screening methods can affect the characters of the aptamers. An aptamer (i.e., GABAS-01) that has great selectivity against GHSA was discovered through five cycles of modified rGO-SELEX. The K_d_ value of GABAS-01 was 1.748 ± 0.227 nM, and the LOD was 16.40 μg/mL. Although the aptamers specific for GHSA have been reported, GABAS-01 has higher sensitivity than previously reported aptamers [38]. This result suggests the potential applications of GABAS-01 as an effective on-site detection probe of GHSA. These properties of GABAS-01 could lead to its use for detection of GHSA in diagnostic fields.

## Figures and Tables

**Table 1:** Aptamer screening condition of each rounds with rGO-SELEX.

Round	ssDNA (pmole)	Binding Condition	Binding Time (min)	Elution Condition	Target Amount (μg)	Elution Time (min)
1	200	1× Binding buffer with 100 μg of rGO and 500 μg of albumin	60	1× PBS	500	60
2						45
3				1.5× PBS		
4						30
5				2× PBS		

**Table 2:** Aptamer sequences after screening with rGO-SELEX.

Name	Sequence	Frequency
GABAS-01	ATGCGGATCCCGCGCGCAGTGCAGGGAGCCGCTCCACGTACGTTGCGCGAAGCTTGCGC	14
GABAS-02	ATGCGGATCCCGCGCGCGGTGGCGAGGGGCCGACGGCGGCTGGGTGCGCGAAGCTTGCGC	9
GABAS-03	ATGCGGATCCCGCGCGGGTCTGAGGAGTGCGGCGGTGCCAGTGAGTGCGCGAAGCTTGCGC	5
GABAS-04	ATGCGGATCCCGCGCCGTGTTAGGCTAGATGTAGAGTTGGTCTGGTGCGCGAAGCTTGCGC	4
GABAS-05	ATGCGGATCCCGCGCGACCAACGGAAGCGCGGCACCACAACGGTGGCGCGAAGCTTGCGC	4
GABAS-06	ATGCGGATCCCGCGCCGAGTCAGTGCGAGGCGCTCCCCTGTCGGTGCGCGAAGCTTGCGC	3
GABAS-07	ATGCGGATCCCGCGCGACTGGACAGGTAATACGGCAGCGGCCGAGGCGCGAAGCTTGCGC	2
GABAS-08	ATGCGGATCCCGCGCGCAATAGGTAAGAATCAGGAGACTGCGTGGGCGCGAAGCTTGCGC	2
GABAS-09	ATGCGGATCCCGCGCTTCTCAAACGCCGGAATGGTTGTTAGTGTGGCGCGAAGCTTGCGC	2
GABAS-10	ATGCGGATCCCGCGCACCTGAAAGCCGCAATGCCAGTGGTCCGTGGCGCGAAGCTTGCGC	1
GABAS-11	ATGCGGATCCCGCGCCGACTACCTTATTTATCCGGGGGAATCCTTGCGCGAAGCTTGCGC	1
GABAS-12	ATGCGGATCCCGCGCGAACATGGAGATGATCACCTTGTGGACTATGCGCGAAGCTTGCGC	1
GABAS-13	ATGCGGATCCCGCGCGAGGTAGGTCCAGGATGAATACGTGGTCTGGCGCGAAGCTTGCGC	1
GABAS-14	ATGCGGATCCCGCGCGCACGATAATTTCCCTTCTCCTGCTGGTCAGCGCGAAGCTTGCGC	1

**Table 3:** Detection of GHSA from buffer and human serum solution.

Detection Method	Spiked (µg/mL)	Measured (µg/mL)	Recovery (%)	RSD (%)
1× PBS	15.625	15.811	101.20	2.52
	31.25	31.535	100.91	1.91
	62.5	62.689	100.30	1.64
	125	125.15	100.12	1.14
Human serum solution diluted by 1× PBS	15.625	16.879	108.03	1.03
	31.25	33.502	107.21	2.70
	62.5	62.937	100.70	1.33
	125	127.34	101.87	1.90

**Table 4:** Detection methods for GHSA used in previous studies.

Detection Method	LOD	Reference
Enzymatic assay-based colorimetric sensor	0.47 mg/mL	[57]
Enzymatic assay-based sensor	0.36 mg/mL	[58]
Graphene-based optical aptasensor	50 μg/mL	[38]
Graphene-based optical aptasensor	16.40 μg/mL	This study
